# Infection and Transmission of Rift Valley Fever Viruses Lacking the NSs and/or NSm Genes in Mosquitoes: Potential Role for NSm in Mosquito Infection

**DOI:** 10.1371/journal.pntd.0001639

**Published:** 2012-05-01

**Authors:** Mary B. Crabtree, Rebekah J. Kent Crockett, Brian H. Bird, Stuart T. Nichol, Bobbie Rae Erickson, Brad J. Biggerstaff, Kalanthe Horiuchi, Barry R. Miller

**Affiliations:** 1 Division of Vector-Borne Diseases, Centers for Disease Control and Prevention, Fort Collins, Colorado, United States of America; 2 Viral Special Pathogens Branch, Division of High-Consequence Pathogens and Pathology, Centers for Disease Control and Prevention, Atlanta, Georgia, United States of America; USAMRIID, United States of America

## Abstract

**Background:**

Rift Valley fever virus is an arthropod-borne human and animal pathogen responsible for large outbreaks of acute and febrile illness throughout Africa and the Arabian Peninsula. Reverse genetics technology has been used to develop deletion mutants of the virus that lack the NSs and/or NSm virulence genes and have been shown to be stable, immunogenic and protective against Rift Valley fever virus infection in animals. We assessed the potential for these deletion mutant viruses to infect and be transmitted by *Aedes* mosquitoes, which are the principal vectors for maintenance of the virus in nature and emergence of virus initiating disease outbreaks, and by *Culex* mosquitoes which are important amplification vectors.

**Methodology and Principal Findings:**

*Aedes aegypti* and *Culex quinquefasciatus* mosquitoes were fed bloodmeals containing the deletion mutant viruses. Two weeks post-exposure mosquitoes were assayed for infection, dissemination, and transmission. In *Ae. aegypti*, infection and transmission rates of the NSs deletion virus were similar to wild type virus while dissemination rates were significantly reduced. Infection and dissemination rates for the NSm deletion virus were lower compared to wild type. Virus lacking both NSs and NSm failed to infect *Ae. aegypti*. In *Cx. quinquefasciatus*, infection rates for viruses lacking NSm or both NSs and NSm were lower than for wild type virus.

**Conclusions/Significance:**

In both species, deletion of NSm or both NSs and NSm reduced the infection and transmission potential of the virus. Deletion of both NSs and NSm resulted in the highest level of attenuation of virus replication. Deletion of NSm alone was sufficient to nearly abolish infection in *Aedes aegypti* mosquitoes, indicating an important role for this protein. The double deleted viruses represent an ideal vaccine profile in terms of environmental containment due to lack of ability to efficiently infect and be transmitted by mosquitoes.

## Author Summary

Rift Valley fever virus is transmitted mainly by mosquitoes and causes disease in humans and animals throughout Africa and the Arabian Peninsula. The impact of disease is large in terms of human illness and mortality, and economic impact on the livestock industry. For these reasons, and because there is a risk of this virus spreading to Europe and North America, it is important to develop a vaccine that is stable, safe and effective in preventing infection. Potential vaccine viruses have been developed through deletion of two genes (NSs and NSm) affecting virus virulence. Because this virus is normally transmitted by mosquitoes we must determine the effects of the deletions in these vaccine viruses on their ability to infect and be transmitted by mosquitoes. An optimal vaccine virus would not infect or be transmitted. The viruses were tested in two mosquito species: *Aedes aegypti* and *Culex quinquefasciatus.* Deletion of the NSm gene reduced infection of *Ae. aegypti* mosquitoes indicating a role for the NSm protein in mosquito infection. The virus with deletion of both NSs and NSm genes was the best vaccine candidate since it did not infect *Ae. aegypti* and showed reduced infection and transmission rates in *Cx. quinquefasciatus*.

## Introduction

Rift Valley fever virus (RVFV), a human and animal pathogen that is endemic in much of Africa, has in recent decades spread to Saudi Arabia, Madagascar and Yemen and has the potential to spread to other parts of the world via transport of infected livestock, humans or mosquitoes or by an act of bioterrorism [1]–[6]. An arthropod-borne member of the *Phlebovirus* genus of the family *Bunyaviridae*, RVFV causes significant outbreaks of severe disease in livestock, including mortality in young animals, fetal deformities and abortion. RVFV infection in humans can result in a self-limiting febrile illness or more severe disease such as retinitis, hepatic necrosis, encephalitis, neurologic deficits or fatal hemorrhagic fever [7]–[9]. The primary maintenance host and source of RVFV initiating disease outbreaks is considered to be mosquitoes in the *Aedes* genus. Mosquitoes in the *Culex* genus are thought to be important in amplification of virus activity during outbreaks. The virus has also been detected in phlebotomine sand flies, *Culicoides* midges, and *Amblyomma* tick species although these infections are not thought to play an important role in the life cycle of the virus or in disease outbreak settings [5], [10]–[13]. In laboratory studies, several North American *Aedes* and *Culex* mosquito species have been shown to be competent vectors of the virus, indicating the potential for establishment of RVFV transmission cycles in North America [14]–[17].

Infection, replication and transmission of an arthropod-borne virus involve complex interactions between the virus and various cells/tissues/organs of the vector. Successful transmission requires that after being ingested in a viremic bloodmeal the virus must enter the epithelial cells of the midgut, replicate and escape from the midgut cells into the hemolymph. This is followed by infection of secondary organs, including the salivary glands, where the virus enters the saliva and can then be transmitted to a new host. Potential barriers in this process have been identified that can block infection, replication and/or transmission of a virus by the mosquito [18], [19]. These include the midgut infection and escape barriers and the salivary gland infection and escape barriers. The presence or absence of these barriers and the degree to which they are effective appears to be influenced by the genetics of both the virus and the vector [18].

The RVFV genome is comprised of three segments of single-stranded, negative sense RNA. The small (S) segment codes for the structural nucleoprotein (NP) and the nonstructural NSs protein, the medium (M) segment encodes the two structural glycoproteins, Gn and Gc, as well as two nonstructural proteins (NSm and NSm-Gn) and the large (L) segment codes for the viral RNA-dependent RNA polymerase. The nonstructural NSs and NSm proteins have been shown to function as virulence factors. The NSs protein has multiple functions that suppress the mammalian host cell antiviral response by inhibiting IFN-β gene transcription, promoting degradation of protein kinase (PKR) and suppressing host transcription [20]–[24]. The RVFV NSm protein plays a role in viral pathogenesis by suppression of virus-induced apoptosis in infected cells although it has been shown to be dispensable for efficient virus growth in cell culture [25]–[27]. To date, little is known regarding the role of the NSs and NSm proteins in the RVFV replication cycle and dissemination and transmission in arthropod vectors.

Historically, a number of different methods have been employed in development of RVFV vaccines, however due to drawbacks associated with currently available vaccines including the necessity for multiple inoculations, abortions/teratologic effects in some vaccinated animals or risk of reversion to virulent phenotype, none of the existing vaccines is approved for veterinary use in North America or Europe [3], [28]. More recently, a reverse genetics methodology has been used to develop recombinant (rRVF) vaccine candidate viruses which contain complete deletions of one or both of the RVFV virulence genes NSs and NSm [29]. These rRVF viruses have been shown to be highly immunogenic and effective at preventing RVFV-associated morbidity and mortality [29]. Additionally, these gene deletions provide the basis for assays to differentiate between vaccinated and naturally infected animals [30].

The purpose of this study was to determine the effects of NSs and NSm gene deletions on infection, dissemination and transmission of these rRVF vaccine candidate viruses in mosquitoes. Results are presented for each of the three deletion mutant rRVF viruses and rRVF-wild type evaluated side-by-side in two mosquito species representing two different genera: *Aedes* (*Stegomyia*) *aegypti* L. and *Culex* (*Culex*) *quinquefasciatus* (Say).

## Materials and Methods

### Viruses and Mosquitoes

Construction of the rRVF viruses has been previously described [25], [29], [31]. Reverse genetics-generated rRVF-wild type (rRVF-wt) and three deletion mutant viruses were used in this study (Table 1). Rescue of rRVF viruses was as previously described [29]. All rescued viruses were fully sequenced as previously described [32]. Virus nomenclature and titers of the Vero E6-2 passage of the viruses are listed in Table 1. Growth curves for each rRVF virus were conducted in Vero E6 cells to determine the optimal virus growth time for bloodmeal preparation. Cell monolayers were infected with virus in Dulbecco's Minimal Essential Medium/2% fetal bovine serum (FBS) with 100 U/mL penicillin and 100 µg/mL streptomycin (DMEM) at a multiplicity of infection (m.o.i.) of 0.1 plaque forming unit (PFU) per cell. Following adsorption for 1 hr at 37°C cells were washed three times with DMEM and then maintained in DMEM at 37°C. Samples were removed daily for 5 days and titers were determined by double overlay plaque titration assay on Vero cells as previously described [33]. Second overlays were added at 3 days post infection (p.i.) and plaques were counted on days 4–7 p.i.

**Table 1:** Reverse genetics-generated RVF viruses used in this study.

Virus Designation	Description	Titer^1^
rRVF-wt	wild type	8.3
rRVF-ΔNSs	NSs gene deleted	8.2
rRVF-ΔNSm	NSm gene deleted	7.0
rRVF-ΔNSs-ΔNSm	NSs and NSm genes deleted	8.0

1 Vero E6 cell, passage 2 titer expressed as log_10_ PFU/ml.

Two mosquito species were used in this study. The *Aedes aegypti* Rexville D mosquito strain used was an isofemale line derived from a population of *Ae. aegypti* collected as larvae in San Juan, Puerto Rico (Rexville) in 1991 [34]. The *Culex quinquefasciatus* Sebring mosquito strain used was originally colonized in Florida in 1998 and has been in colony at the CDC in Fort Collins since 2004 [35]. The species identity of the *Cx. quinquefasciatus* colony was verified by examination of genetalia and by HotAce PCR [36]–[38]. These species were selected because they are found in Africa, where the RVFV candidate vaccines being tested will primarily be used, because of their availability as colonized populations and because their vector competence for RVFV has been previously characterized [13], [15], [16], [39].

### Oral Infection of Mosquitoes

Multiple blood-feeding experiments were undertaken. Each experiment utilized freshly prepared virus due to an observed reduction in infection rates when frozen virus was used in bloodmeals: in a separate experiment we observed only a 10% (n = 19) rate of infection in *Ae. aegypti* mosquitoes fed a bloodmeal containing frozen rRVF-wt virus compared to 63% (n = 32) with freshly harvested virus.

For each experiment, adult 7- to 10-day old mosquitoes were placed in pint cartons (approx. 50–100 mosquitoes per carton) and starved for 24 hours prior to feeding. Artificial virus-laden bloodmeals were prepared using fresh virus grown in Vero E6 cells as described above. Virus was harvested 2–3 days after infection depending on growth curve results for each virus (data not shown). Infected cell culture supernatant was clarified by centrifugation at 10 K rpm, 4°C for 10 min. Defibrinated chicken blood (Colorado Serum Co., Denver, CO) was washed 3 times with 1 volume of ice-cold phosphate buffered saline and centrifuged at 3 K rpm, 4°C for 3 min after each wash. Two parts clarified virus were mixed with 2 parts washed blood and 1 part FBS/10% sucrose. The bloodmeal was heated to 37°C and offered to mosquitoes using cotton balls that were soaked in the bloodmeal and applied to the mesh top of the mosquito cartons. Mosquitoes were allowed to feed for 30 min at 28°C/95% humidity after which the bloodmeal was removed. Fully engorged mosquitoes were collected, double-caged and held for 14 days at 28°C/95% humidity with 5% sugar water. Three engorged mosquitoes were immediately removed for each virus and titrated to determine the amount of virus ingested (input virus titer).

### Mosquito Testing

Twenty-five to fifty mosquitoes from each virus group were tested for virus transmission at 14 days post exposure by collection of saliva as previously described [40]. Briefly, specimens were anesthetized by chilling at −20°C for 1 min, then, inside a glove box, wings were removed and the proboscis of each specimen was inserted into a capillary tube containing 5 µL immersion oil and saliva collected for 20 min. The tip of each capillary tube was clipped off into a microfuge tube containing 250 µL DMEM/10% FBS, tubes were centrifuged 5 min at 5000 rpm at 4°C to draw the oil out of the capillary tube and titers were determined as described above [33]. Observation of one or more viral plaques was considered a positive result. Following saliva collection, individual mosquito bodies were stored at −80°C. Additional day 14 mosquitoes were stored at −80°C and were tested only for dissemination and/or infection status. Mosquitoes were subsequently tested for virus dissemination by head squash and immunofluorescence assay (IFA) as previously described, using mouse-anti RVFV strain ZH501 hyperimmune ascitic fluid diluted 1∶2500 as the primary antibody and goat-anti-mouse IgG-Alexa488 (Invitrogen, Baltimore, MD) diluted 1∶2000 as the secondary antibody conjugate [40]. Observation of specific fluorescence as compared to uninfected controls was considered a positive result. The infection status of mosquitoes was determined by trituration of bodies in 2 mL conical microcentrifuge tubes with 1 mL BA-1 medium (1× medium 199 with Earle's salts, 1% bovine albumin, 100 U/mL penicillin, 100 µg/mL streptomycin, and 1 µL/mL amphotericin B) and one copper BB per tube using a Qiagen Tissuelyser (Qiagen, Valencia, CA). Triturated mosquito preparations were clarified by centrifugation at 9 K rpm/4°C for 10 min followed by plaque titration of the clarified supernatant on Vero cells as above.

Virus was isolated and sequenced from selected mosquitoes at 14 days post-exposure as follows. Viral RNA was extracted either from triturated mosquito supernatant or from a Vero cell amplification of mosquito supernatant (25 µL mosquito supernatant grown 3 days in a T25 flask of Vero cells) using the QIAamp viral RNA kit (Qiagen). RT-PCR was performed using the Titan One-Step RT-PCR kit (Roche, Indianapolis, IN). Products were agarose gel purified and sequenced using the BigDye Terminator v3.1 Ready Reaction Cycle Sequencing mix (Applied Biosystems, Foster City, CA). Reactions were purified using the BigDye Xterminator Purification kit (Applied Biosystems) and analyzed on an ABI 3130 Genetic Analyzer (Applied Biosystems).

### Statistical Analysis

Linear regression methods were used to compare (log_10_-transformed) titers among the virus constructs, while logistic regression was used to compare their infection, dissemination, and transmission rates. Wald 95% confidence intervals were computed for parameters of interest, and likelihood ratio tests were used to compare models. Because the data have cases for which all individuals in a virus test group were either negative or positive, we use Firth's penalized likelihood adjustment to the estimating score equations, as detailed in Heinze and Schemper (2002) and Heinze and Puhr (2010) and implemented in R (www.r-project.org) in the package *logistf* [41], [42]. All analyses were conducted in R version 2.11.1 (www.r-project.org). Confidence intervals for the differences of virus effects were adjusted for multiple comparisons in both normal and logistic models using the methods described in Hothorn et al. (2008) [43].

Due to the necessity of using freshly grown, and therefore untitrated, virus in the oral mosquito feeds, the standard regression methods for the body titers and infection rates were augmented to adjust for the unknown amount of virus taken up during the feedings by using the information collected from the mosquitoes fed concurrently and stored just after feeding (input virus titer). This was necessary because the titers for the different viruses varied between each virus stock preparation. Although significant results were found, we cannot rule out that this variation in the virus titers may have affected the results in a manner that cannot be accounted for by the statistical analysis. To summarize the approach, we treated the unknown amount of virus taken up by the test mosquitoes as missing data, represented in the linear models as a simple, continuous random effect and in the logistic models as a continuous, random offset. We then used the estimated, predictive normal distributions of the concurrently fed individuals' input virus log_10_-titer measurements from the corresponding virus to impute values for the unknown virus uptake of the test individuals. For each individual we generated 100 such imputations, fit regression models to each of these “completed” datasets, and averaged the parameters from the resulting model fits. Statistical comparisons and tests, confidence intervals and p-values incorporated both the modeling uncertainty and the imputation uncertainty; see Little and Ruben (2002) for details related to analysis of missing data and incorporation of imputation error [44].

For the dissemination and transmission rates, Fisher's exact tests were used to test for an overall difference, and pairwise comparisons among viruses were made using score confidence intervals for the differences. The Bonferroni adjustment was used to account for the multiple comparisons.

## Results

Viral RNA from 25 randomly selected infected mosquitoes representing both species and all four viruses was sequenced and compared to the nucleotide sequence of the bloodmeal viruses; no differences were observed. Results from *Ae. aegypti* and *Cx. quinquefasciatus* experiments are listed in Tables 2 and 3, respectively. Dissemination rates in these tables are calculated in two ways: 1) D_e_ = number positive divided by number exposed to virus and 2) D_i_ = number positive divided by number infected. Similarly, transmission rates are shown as 1) T_e_ = number positive divided by number exposed to virus and 2) T_d_ = number positive divided by number disseminated. Where data from two separate experiments are presented in Tables 2 and 3 we have combined and summarized the results in the text below as noted.

**Table 2:** Replication of rRVF mutant viruses in *Aedes aegypti* mosquitoes.

						Dissemination rates				Transmission rates			
				Infection rate^1^		# pos/# exposed^2^		# pos/# infected^3^		# pos/# exposed^4^		# pos/# disseminated^5^	
Virus	Experiment	Bloodmeal titer^6^	Avg (sd) day 0 titer^7^	% positive (n)		% positive (n)		% positive (n)		% positive (n)		% positive (n)	
rRVF-wt	1	6.9	4.2 (0.3)	–	nd^8^	83.9	(31)	–	nd	–	nd		nd
rRVF-wt	2	6.9	4.2 (0.1)	62.5	(32)	56.3	(32)	90.0	(20)	46.9	(32)	83.3	(18)
rRVF-ΔNSs	1	6.9	4.0 (0.2)	–	nd	34.0	(50)*	–	nd	–	nd		nd
rRVF-ΔNSs	2	8.3	5.3 (0.3)	88.9	(36)	44.4	(36)*	50.0	(32)*	33.3	(36)	75.0	(16)
rRVF-ΔNSm	1	6.9	4.1 (0.1)	11.1	(45)*	2.2	(45)*	20.0	(5)*	2.2	(45)*	100.0	(1)
rRVF-ΔNSm	2	6.2	3.3 (0.1)	0	(84)*	0	(84)*	–		0	(50)*	–	
rRVF-ΔNSs-ΔNSm	2	7.0	4.0 (0.2)	0	(75)*	0	(84)*	–		0	(50)*	–	

1 Number of mosquitoes containing detectable virus by plaque assay divided by number of mosquitoes exposed to infectious bloodmeal.

2 D_e_ = number of mosquitoes with RVFV antigen in head tissues divided by number of mosquitoes exposed to infectious bloodmeal.

3 D_i_ = number of mosquitoes with RVFV antigen in head tissues divided by number of infected mosquitoes.

4 T_e_ = number of mosquitoes with positive saliva by plaque assay divided by number of mosquitoes exposed to infectious bloodmeal.

5 T_d_ = number of mosquitoes with positive saliva by plaque assay divided by number of mosquitoes with disseminated infection.

6 Titer expressed as log_10_ PFU/mL.

7 n = 3, titer expressed as log_10_ PFU/ml; avg is log_10_ geometric mean; sd is standard deviation.

8 nd = not done.

*: value differs significantly from rRVF-wt values; see Table S2 for statistical analysis results.

**Table 3:** Replication of rRVF mutant viruses in *Culex quinquefasciatus* mosquitoes.

						Dissemination rates				Transmission rates			
				Infection rate^1^		# pos/# exposed^2^		# pos/# infected^3^		# pos/# exposed^4^		# pos/# disseminated^5^	
Virus	Experiment	Bloodmeal titer^6^	Avg (sd) day 0 titer^7^	% positive (n)		% positive (n)		% positive (n)		% positive (n)		% positive (n)	
rRVF-wt	3	7.9	4.3 (0.2)	94.3	(35)	9.9	(131)	7.1	(28)	6.7	(30)	100.0	(2)
rRVF-wt	4	8.2	5.5 (0.4)	95.7	(25)	16.0	(25)	16.7	(24)	4.0	(25)	25.0	(4)
rRVF-ΔNSs	3	8.5	5.8 (0.2)	94.3	(35)	7.1	(113)	11.4	(35)	6.7	(30)	66.7	(3)
rRVF-ΔNSm	3	6.5	3.2 (0.2)	11.4	(35)*	0.8	(119)*	25.0	(4)	0	(30)	0	(1)
rRVF-ΔNSs-ΔNSm	4	8.3	6.2 (0.2)	66.0	(50)*	10.0	(50)	15.2	(33)	4.0	(50)	40.0	(5)

1 Number of mosquitoes containing detectable virus by plaque assay divided by number of mosquitoes exposed to infectious bloodmeal.

2 D_e_ = number of mosquitoes with RVFV antigen in head tissues divided by number of mosquitoes exposed to infectious bloodmeal.

3 D_i_ = number of mosquitoes with RVFV antigen in head tissues divided by number of infected mosquitoes.

4 T_e_ = number of mosquitoes with positive saliva by plaque assay divided by number of mosquitoes exposed to infectious bloodmeal.

5 T_d_ = number of mosquitoes with positive saliva by plaque assay divided by number of mosquitoes with disseminated infection.

6 Titer expressed as log_10_ PFU/mL.

7 n = 3, titer expressed as log_10_ PFU/ml; avg is log_10_ geometric mean; sd is standard deviation.

*: value differs significantly from rRVF-wt values; see Table S2 for statistical analysis results.

### *Ae. aegypti*

Replication of the rRVF-wt and deletion mutant viruses in *Ae. aegypti* mosquitoes is summarized in Table 2 with statistical analysis results data available in Table S1. The infection rate observed for the rRVF-ΔNSs virus (32/36, 88.9%) did not differ significantly from that observed for the rRVF-wt virus (20/32, 62.5%), although the average body titer at 14 days post-exposure for individuals infected with rRVF-ΔNSs (3.7 log_10_ PFU/mL) was significantly lower than for rRVF-wt (5.4 log_10_ PFU/mL) (P<0.01, data not shown). Dissemination rates for the rRVF-ΔNSs virus (D_e_, from two experiments combined = 33/86, 38.4%, and D_i_ = 16/32, 50%) were significantly lower than for rRVF-wt (D_e_ combined = 44/63, 69.8% and D_i_ = 18/20, 90%), while transmission rates for rRVF-ΔNSs (T_e_ = 12/36, 33.3% and T_d_ = 12/16, 75%) and rRVF-wt (T_e_ = 15/32, 46.9% and T_d_ = 15/18, 83.3%) did not differ significantly. RVFV antigen was found to be similarly distributed throughout head tissues by IFA testing of individuals with disseminated rRVF-wt and rRVF-ΔNSs infections (data not shown).

The *Ae. aegypti* infection rate for the rRVF-ΔNSm virus (5/129, 3.9%, combined) was significantly less than for rRVF-wt (20/32, 62.5%). The rRVF-ΔNSm infection rate in experiment 1 (5/45, 11.1%) was higher than that of experiment 2 (0/84, 0%), most likely due to the higher experiment 1 bloodmeal titer, although when calculated individually both rates were significantly less than that of rRVF-wt. The average body titer of rRVF-ΔNSm-infected mosquitoes (1.9 log_10_ PFU/mL) at 14 days post-exposure was significantly less than that of mosquitoes infected with rRVF-wt (5.4 log_10_ PFU/mL) (P<0.01, data not shown). The dissemination rates for rRVF-ΔNSm (D_e_ combined = 1/129, 0.8% and D_i_ = 1/5, 20%) were significantly less than rRVF-wt (D_e_ combined = 44/63, 69.8% and D_i_ = 18/20, 90%). The transmission rate for rRVF-ΔNSm (T_e_ combined = 1/95, 1.1%,) was significantly less than that of rRVF-wt (T_e_ = 15/32, 46.9%) when calculated as T_e_ (number positive/number exposed); when calculated as T_i_ (number positive/number disseminated) the transmission rate did not differ significantly from rRVF-wt. Out of 129 *Ae. aegypti* mosquitoes that fed on a bloodmeal containing the rRVF-ΔNSm virus, five became infected and one developed a disseminated infection; this individual was also found to be transmission-positive. The distribution of RVFV antigen in head tissues of this individual did not appear to differ from that of rRVF-wt (data not shown). Full length sequencing of virus isolated from this individual revealed no genetic differences compared to the virus in the blood meal.

None of the 75 *Ae. aegypti* mosquitoes exposed to rRVF-ΔNSs-ΔNSm were found to be infection-, dissemination- or transmission-positive.

### *Cx. quinquefasciatus*

Replication of the rRVF-wt and deletion mutant viruses in *Cx. quinquefasciatus* mosquitoes is summarized in Table 3 with statistical analysis data presented in Table S2. Similarly high infection rates were observed for rRVF-wt (57/60, 95%, combined) and rRVF-ΔNSs (33/35, 94.3%), while significantly lower rates were observed for the constructs containing the NSm deletion [rRVF-ΔNSm (4/35, 11.4%) and rRVF-ΔNSs-ΔNSm (33/50, 66%)]. There were no significant differences in the dissemination or transmission rates of the rRVF-ΔNSs or rRVF-ΔNSs-ΔNSm viruses compared to the rRVF-wt virus. When calculated as D_e_ (number positive/number exposed), the dissemination rate for the rRVF-ΔNSm virus (D_e_ = 1/119, 0.8%) was significantly less than that of rRVF-wt (D_e_ combined = 17/156, 10.9%), however, the dissemination rate calculated as D_i_ (number positive/number infected) was not significantly different and there were no significant differences in transmission rates between these viruses. IFA testing demonstrated the presence of RVFV antigen distributed throughout head tissues from *Cx. quinquefasciatus* individuals with a disseminated infection; no qualitative differences were observed between tissues infected with the deletion mutant viruses and rRVF-wt (data not shown). Average body titers of individuals with disseminated infections ranged from 4.4–6.1 log_10_ PFU/mL, while titers in individuals with undisseminated infections ranged from 1.0–4.3 log_10_PFU/mL. These values are similar to those reported for *Cx. pipiens* mosquitoes by Turell et al. [14].

## Discussion

We report the *in vivo* infection, dissemination and transmission characteristics of several recombinant RVF viruses lacking the entire coding regions of the NSs and/or the NSm genes and demonstrate the critical role of the NSm gene for infection and transmission in two mosquito species that exhibit different capacities for transmitting RVFV. *Ae. aegypti* has been shown to be a moderately competent vector of RVFV, although it has not been shown to be a vector in nature [13], [39]. *Cx. quinquefasciatus* is a potential vector of RVFV in nature, although laboratory studies have shown it to be a less efficient vector than *Ae. aegypti.* [5], [13], [39], [45]. These species were selected based on this difference in vector competence, because both are found in Africa where the candidate vaccine viruses tested here will primarily be used and because both have been colonized for use in laboratory investigations.

Observed rates of dissemination of rRVF-wt in *Ae. aegypti* were much greater than those in *Cx. quinquefasciatus* mosquitoes in our study. At 14 days post-exposure, 90% of rRVF-wt-infected *Ae. aegypti* individuals had titers greater than the average input virus titer. This is in marked contrast to the *Cx. quinquefasciatus* mosquitoes, where at 14 days post-exposure only 10.5% had a rise in body titer that was greater than the input virus titer and 35% of individuals with detectable virus at 14 days post-exposure had body titers ≤2.0 _log10_ PFU/mL. Additionally, dissemination rates for rRVF-wt in *Cx. quinquefasciatus* were low (≤16.7%) (Table 3). These observations support the hypothesis that a midgut infection and/or midgut escape barrier is responsible for the lower vector competence of this species compared to *Aedes* species for RVFV [13], [15], [16].

The recombinant viruses tested comprised three groups: those with a deletion of the NSs gene from the S segment of the virus, those with a deletion of the NSm gene from the M segment, and those with both the NSs and NSm genes deleted. Recombinant RVFV lacking the NSs gene has been shown to maintain the virulence and growth characteristics of the wild type virus in mammalian cell culture, and *in vivo* testing demonstrated it to be highly attenuated, immunogenic and protective against challenge with wild type virus making it a potential vaccine candidate [29]. RVFV Clone 13, an attenuated clone containing a deletion of 70% of the NSs gene, has been shown to exhibit a lower infection rate in *Cx. quinquefasciatus* mosquitoes compared to wild type RVFV ZH548, while no difference was observed in *Ae. vexans* mosquito infection rates [46]. In our study, deletion of the NSs gene alone did not significantly affect rates of infection or transmission compared to rRVF-wt in either *Ae. aegypti* or *Cx. quinquefasciatus* mosquitoes although the dissemination rate for rRVF-ΔNSs was significantly lower than rRVF-wt in *Ae. aegypti*.

Recombinant RVFV lacking the NSm gene exhibits efficient replication in cell culture and although *in vivo* studies have demonstrated this mutant virus to be highly immunogenic it is only partially attenuated relative to the wild type virus, retaining the ability to cause lethal hepatic or neurologic disease in a minority of infected animals [29], [31]. In the current study, deletion of the NSm gene significantly reduced infection rates in both mosquito species tested. In *Ae. aegypti* mosquitoes, dissemination and transmission rates were also significantly reduced suggesting an important role for the NSm proteins in this species.

Given the characteristics of RVFV mutant viruses individually lacking the NSs or NSm genes, Bird, et al., have hypothesized that combining these deletions in a single mutant virus would generate a stable, attenuated, immunogenic vaccine virus [29]. Results of *in vitro* and *in vivo* studies characterizing the double NSs and NSm deleted recombinant virus suggest that this hypothesis is correct [29]. The double deletion virus grows efficiently in cell culture and in animals this virus is highly attenuated, immunogenic and confers protective immunity from wild type virus challenge [29].

We observed that deletion of the NSs and NSm genes in combination affected RVFV growth differently in the two mosquito species tested. Deletion of both the NSs and NSm genes had a pronounced effect in *Ae. aegypti* mosquitoes; none of the *Ae. aegypti* that ingested a bloodmeal containing the rRVF-ΔNSs-ΔNSm virus became infected (n = 75). In contrast, infection with rRVF-ΔNSs-ΔNSm of the less competent vector, *Cx. quinquefasciatus*, was significantly reduced compared to rRVF-wt, although to a lesser degree than in *Ae. aegypti*, and no significant differences in dissemination or transmission rates were observed. It was apparent, however, that in *Cx. quinquefasciatus* the additional deletion of the NSm gene reduced the infection rate of the double deletion virus, rRVF-ΔNSs-ΔNSm, (33/50, 66.0%) compared to the rRVF-ΔNSs single deletion virus (33/35, 94.3%) (Table 3). The *Cx. quinquefasciatus* rRVF-ΔNSs-ΔNSm infection rate was higher than the rRVF-ΔNSm rate, however this is most likely due to the higher titer of the rRVF-ΔNSs-ΔNSm bloodmeal.

*In vivo* studies by Bird, et al., showed no detectable post-vaccination viremia in n = 20 rats [29] and more recently in n = 42 sheep inoculated with the double deletion virus [47]. These results, coupled with our observation that in the more competent *Ae. aegypti* vector a bloodmeal titer of 7.0 log_10_ PFU/mL did not result in infection of mosquitoes with this virus, suggest an extremely low likelihood that mosquitoes could acquire an infectious dose of virus from feeding on a vaccinated animal. This is important since, due to the segmented nature of the RVFV genome, the potential exists for reassortment and therefore reversion of mutations in a mosquito that has acquired both vaccine and wild type strains of the virus from feeding on multiple hosts [48]. The deletion of genes from multiple segments of the virus genome in the rRVF-ΔNSs-ΔNSm vaccine candidate virus adds an additional safeguard against this mechanism of reassortment-driven reversion to wild type.

The results of this study demonstrate that deletion of the RVFV NSm gene alone or in combination with the NSs gene significantly affected the replication kinetics of the virus in the mosquito species tested, particularly in *Ae. aegypti*. The combined deletion of both gene regions resulted in the greatest attenuation of RVFV replication in these mosquitoes, suggesting that the rRVF-ΔNSs-ΔNsm virus is an acceptable vaccine candidate with little possibility of environmental contamination due to the lack of efficient infection and transmission in mosquitoes.

The RVFV NSm has been demonstrated to function as a suppressor of virus-induced apoptosis in mammalian cells in culture although a similar role has not been demonstrated in arthropod cells [26]. However, NSm has also been shown to be non-essential for replication in cultured mammalian cells suggesting it may have a more significant function in the infection of insect vectors involved in amplification and transmission of RVFV in nature [25], [27]. In our study, the reduced infection rates observed in both *Ae. aegypti* and *Cx. quinquefasciatus* species and the diminished whole body virus titers of infected *Ae. aegypti* mosquitoes suggest a possible role for NSm in modulation of a mosquito midgut infection barrier. Additionally, the reduction in rates of dissemination and transmission in *Ae. aegypti* indicate that NSm may also function as a suppressor of a midgut escape barrier. Although the mechanisms of these barriers are not understood, it is apparent that the genetic traits of the virus as well as those of the mosquito host species influence the infection, dissemination and transmission of arboviruses [18]. The rRVF-ΔNSm deletion mutant will be a valuable tool in future studies to elucidate the mechanisms of RVFV infection and transmission in mosquito vectors.

## Supporting Information

Table S1 ***Aedes aegypti*** **statistical analysis results for data in** **Table 2** **.**
(XLS)

Table S2 ***Culex quinquefasciatus*** **statistical analysis results for data in** **Table 3** **.**
(XLS)
